# Mechanisms of immune suppression by myeloid-derived suppressor cells: the role of interleukin-10 as a key immunoregulatory cytokine

**DOI:** 10.1098/rsob.200111

**Published:** 2020-09-16

**Authors:** Mahmoud Mohammad Yaseen, Nizar Mohammad Abuharfeil, Homa Darmani, Ammar Daoud

**Affiliations:** 1 Department of Biotechnology and Genetic Engineering, Jordan University of Science and Technology, Irbid 22110, Jordan; 2 Department of Applied Biology, Faculty of Science and Arts, Jordan University of Science and Technology, Irbid 22110, Jordan; 3 Department of Internal Medicine, Faculty of Medicine, Jordan University of Science and Technology, Irbid 22110, Jordan

**Keywords:** STAT3, PD-1/PD-L1, S100A8/A9, natural killer cells, macrophages, dendritic cells

## Abstract

Chronic immune activation and inflammation are unwanted consequences of many pathological conditions, since they could lead to tissue damage and immune exhaustion, both of which can worsen the pathological condition status. In fact, the immune system is naturally equipped with immunoregulatory cells that can limit immune activation and inflammation. However, chronic activation of downregulatory immune responses is also associated with unwanted consequences that, in turn, could lead to disease progression as seen in the case of cancer and chronic infections. Myeloid-derived suppressor cells (MDSCs) are now considered to play a pivotal role in the pathogenesis of different inflammatory pathological conditions, including different types of cancer and chronic infections. As a potent immunosuppressor cell population, MDSCs can inhibit specific and non-specific immune responses via different mechanisms that, in turn, lead to disease persistence. One such mechanism by which MDSCs can activate their immunosuppressive effects is accomplished by secreting copious amounts of immunosuppressant molecules such as interleukin-10 (IL-10). In this article, we will focus on the pathological role of MDSC expansion in chronic inflammatory conditions including cancer, sepsis/infection, autoimmunity, asthma and ageing, as well as some of the mechanisms by which MDSCs/IL-10 contribute to the disease progression in such conditions.

## 1. Introduction

Inflammatory immune responses are essential for combating infections and malignancies; however, it is important to remember that chronic inflammation could lead to tissue damage and worsen the pathological condition [1–4]. Therefore, controlling such immune responses is of central importance to avoid such unwanted consequences. Paradoxically, shifting the inflammatory to anti-inflammatory immune responses is considered to be a hallmark in the pathogenesis of many inflammatory conditions, including cancer and chronic infections. This is especially because mediating anti-inflammatory responses can limit specific anti-cancer/infection immune responses that, in turn, lead to disease persistence. Hence, it is essential to realize that restoring the balance between inflammatory and anti-inflammatory responses (i.e. immune homeostasis) is a key clue to control chronic inflammatory pathological conditions. To this end, understanding the mechanisms by which such shifts occur in pathological conditions are essential to pave the way to achieve immune homeostasis.

A large body of evidence shows that such immune shifts, at least in part, are driven by a heterogeneous population of immune cells called ‘myeloid-derived suppressor cells' (MDSCs). These cells are innate immune cells of myeloid origin with potent immunosuppressive capabilities, and are known to expand and accumulate in many inflammatory pathological conditions. Expansion of MDSCs is mediated by the activation of different transcription and regulatory factors. These include signal transducer and activator of transcription (STAT3), cyclic adenosine 3′,5′-monophosphate/mitogen-activated protein kinase (cAMP/MAPK), interferon related factor-8 (IRF-8), CCAAT/enhancer-binding protein-β (C/EBP*β*), nuclear factor I-A (NFIA), hypoxia-inducible factor-1 (HIF-1) and retinoblastoma protein 1 (RB1) among others. Activation or repression of such factors was shown to determine the ultimate fate of MDSC differentiation into a distinct population [5–12]. Another important point to be mentioned here is that MDSC expansion is totally governed by the inflammatory microenvironment. In other words, cytokines such as interferon-γ (IFN-γ), interleukin (IL)-β, IL-6, IL-4/IL-13, tumour-necrosis factor-α (TNF-α) or other molecules such as toll-like receptors (TLRs), and prostaglandin E2 (PGE2) activate specific cellular signalling pathway(s) (e.g. STAT1, STAT3, STAT6, cyclooxygenase (COX) and NF-κB) depending on their interaction with the corresponding receptor(s) on the surface myeloid cells (reviewed in [13–15]). Ultimately, these interactions promote the expansion of myeloid cells with suppressive capabilities, namely MDSCs.

In recent years, it has become clearly evident that MDSCs play a major role in the pathology of cancer and many other disorders. It is important to note that most of our knowledge about MDSCs stem from cancer studies. Hence, it will not be surprising to see an emphasis on cancer in the following discussion. Generally speaking, in cancer, MDSCs have a triple pathological role: (i) they suppress specific anti-tumour adaptive immune responses that in turn facilitate tumour growth [16,17]; (ii) they themselves enhance the growth of already established tumours by secreting different molecules [18,19]; and (iii) they could provide a spark to the initiation of tumorigenesis at the inflammatory microenvironment [20–23]. As such, in the following discussion, wherever cancer is mentioned, we will be hovering in the orbit of these three roles.

In fact, the primary target immune cell population that are inhibited by MDSCs are T cells. MDSCs exert their suppressive effects on T cells via different ways, including inhibition of T cell activation and proliferation, inducing T cells anergy, depletion of T-cells by apoptosis, and blocking their homing, etc. through various complex mechanisms that have been recently reviewed in many published articles [24–27]. These mechanisms can be categorized into five major categories: (i) secretion and expression of immunosuppressor molecules such as interleukin-10 (IL-10), tumour growth factor-β (TGF-β), reactive oxygen species (ROS), nitrogen oxygen species (NOS), programmed cell death-1 (PD-1), programmed cell death ligand-1 (PD-L1) and cytotoxic T-lymphocyte-associated protein 4 (CTLA-4); (ii) depletion of metabolites required for T cell functions; (iii) manipulating the expression of chemotactic molecules and ligands that govern the homing of T-cells; (iv) induction of immune suppressor cells such as T regulatory (Treg) cells; and (v) manipulating the metabolism of adenosine by inducing the expression of ectoenzymes [24–27]. However, our exploration will not be restricted to T cells alone but will also be extended to include other immune cells, so that the scattered puzzle pieces can be gathered, especially because the inflammatory microenvironment is of a complex nature.

IL-10 is a potent immunoregulatory (anti-inflammatory) cytokine that is encoded by *IL10* gene in humans. IL-10 is produced by different types of immune cells including monocytes/macrophages (M2 monocytes), dendritic cells, natural killer cells, mast cells, B cells and T cells (type 2 CD4^+^ T-helper cells, Treg cells, and a subset of CD8^+^ T cells), as well as MDSCs [28–30]. Importantly, under certain circumstances, IL-10 has pro-inflammatory activity as well, highlighting its pleiotropic effects [31]. However, the positive association between disease progression and the blood levels of IL-10 in several types of cancer indicates that it has an immunosuppressive effect in cancer [32], and it could be directly, or indirectly, involved in the pathogenesis of such pathological conditions (as will be discussed later). This is at least because the production of IL-10 during inflammatory conditions, such as cancer and infectious diseases, has been shown to inhibit the inflammatory immune responses mediated by different immune cells. The latter include: type-1T helper cells (Th1); natural killer (NK) cells; classically activated macrophages; and myeloid-derived dendritic cells, all of which are essential to initiate type-1 immune responses, namely anti-tumour/infection immune responses (which will be discussed later).

At present, there is a remarkable and growing interest in studying the role of MDSC and IL-10 in chronic inflammatory conditions. Furthermore, the absence of a specific review article that addresses this topic during the past decade, has encouraged us to address the mechanisms by which MDSC/IL-10 can suppress immune responses and facilitate disease progression in different pathological conditions. However, before beginning, we will introduce the reader into some basic concepts about MDSC.

## 2. A glance at MDSCs

MDSCs are a heterogeneous population of myeloid cells, with two main MDSC populations that have been identified in humans, non-human primates and mice, according to their morphology and phenotype. MDSCs that are similar to monocytes in phenotype and morphology and have suppressive activity are called monocytic-MDSCs (M-MDSCs), while those that are similar to polymorphonuclear neutrophils (PMN) in morphology and have suppressive capabilities are called PMN-MDSCs. Under normal conditions, such cells are kept at very low levels [33–40] and to date, the function of MDSCs under normal conditions is not yet established, with some findings reported here and there [41–44]. The reasons behind this lack of information may stem from the fact that MDSCs were originally described only in pathological conditions, mainly in cancer and subsequently in different diseases [33–37,45–53], leading to the idea that MDSCs are usually pathologically activated cells. Furthermore, the similarities in phenotype between M-MDSCs and PMN-MDSCs and normal counterpart cells, namely monocytes and neutrophils, respectively, have also hampered the identification of MDSCs in healthy hosts. In other words, studies have shown that the phenotypes of M-MDSCs and PMN-MDSCs in mice are indistinguishable from normal mouse monocytes and neutrophils, respectively [54]. This is also the case with human neutrophils which cannot be distinguished from PMN-MDSCs based on phenotype alone, and thus practically these cells could not be detected in healthy subjects, especially, before the discovery of the potential candidate markers LOX-1 and SPARC which seem to be exclusively expressed on PMN-MDSCs but not on M-MDSCs or normal neutrophils [54,55]. Furthermore, before unifying the term MDSCs in 2007, there was an inconsistency in MDSCs nomenclature in that these cells were frequently called ‘immature myeloid cells' or ‘myeloid suppressor cells'. The latter terms are not accurate enough to exclusively define such cell population, explaining why other cell populations which lacked the features (mentioned below) that define MDSCs were defined as MDSCs [56–60].

Although the term ‘myeloid-derived suppressor cells' may be more accurate than the terms mentioned earlier, recent advances have shown that this term should also be reevaluated. This is because it can be used to describe only a proportion of the cells, which exhibit all the following features at once, namely, they are of myeloid origin, immature and they are functional having immunosuppressive activity, (reviewed in [15]). In the context of the latter property, recent studies have also identified mature MDSCs with an activated state in addition to the immature MDSC population. Thus, the MDSC population is thought to comprise of a mixture of mature and immature cells. Therefore, the immaturity feature should not be exclusive to MDSCs anymore [15]. On the other hand, from the function point of view, healthy MDSCs isolated from normal individuals (or as we prefer to call them ‘MDSC-like cells' because of the similarities in phenotype with pathologically activated MDSCs) are not immunosuppressive, or at least have much lower suppressive capabilities than pathologically activated ones [41,42,61]. Still, pathologically activated MDSCs greatly vary in their suppressive capabilities (i.e. M-MDSCs have a greater suppressive power than PMN-MDSCs on a ‘per cell' basis [62–64]). Additionally, studies have shown that the suppressive function of pathologically activated MDSCs *in vivo* is limited to the inflammatory microenvironment (site), as declared by Haverkamp *et al*. [65], suggesting that MDSCs could behave normally or abnormally according to the stimuli specific to the milieu (i.e. the tumour microenvironment enhances MDSC immunosuppressive responses). As such, it is not surprising that several studies have also documented that normal monocytes from healthy individuals can acquire the phenotype and the suppressive activity of M-MDSCs upon exposure to tumour cells or a specific microenvironment where certain cytokines such as IL-10, or prostaglandin E2 ‘PGE2' play a role in this transition [66–69]. A similar scenario has also been suggested for the differentiation of neutrophils to PMN-MDSCs [70–72]. Moreover, emerging evidence indicates that MDSCs could express pro-inflammatory functions in certain pathological conditions such as autoimmune disorders [73–75]. It is becoming clear that myeloid-derived cells that express pro-inflammatory activity must not be described as immunosuppressor cells or MDSCs anymore, simply because this is a contradiction. We therefore suggest a new nomenclature for such cells to remove this ambiguity, namely myeloid-derived pro-inflammatory cells (MDPCs) [15].

Taken together, these findings, to a certain extent, explain why the characterization and the function of healthy MDSCs have not been studied in a similar way to that of the pathologically activated MDSCs. Furthermore, the view that we already have about MDSCs becomes more complicated, underscoring the need to characterize MDSCs into pathologically activated and non-pathologically activated cells. In addition, there is a need to reevaluate the term MDSC, especially after the identification of mature MDSCs and pro-inflammatory MDSCs.

## 3. The pathological roles of MDSCs

Undeniably, the main striking function of MDSCs is that they have potent immunosuppressive properties. T cells are the main target cell population for the immunosuppressive activity mediated by MDSCs, and to a lesser extent other immune cell populations, such as NK cells, natural killer T (NKT) cells, M*Φ* and dendritic cells (DCs) [16,76–80]. Several studies have shown that PMN-MDSCs represent the predominant immunosuppressive population in blood and at tumour site(s) of most cancer types, accounting for more than 80% of the total MDSCs present [13,81–83]. Yet, on a per cell basis, as previously mentioned, the suppressive activity of M-MDSCs on T cells is much greater than on PMN-MDSCs [62–64]. Importantly, if the massive number of PMN-MDSCs is taken into account, then one could argue that the overall suppressive effect mediated by MDSCs *in vivo* should be driven by the PMN-MDSC population. Nonetheless, studies have shown that the depletion of PMN-MDSCs did not alter tumour incidence, suggesting that M-MDSCs are the major immunosuppressor cells *in vivo* [84]. However, additional investigations on both cancer related and other unrelated pathological conditions are needed to shed some more light on these findings.

It is becoming apparent that the role that MDSCs play in cancer is not restricted to suppression of anti-tumour immune responses which in turn facilitate tumour growth and metastasis, rather they could be directly involved in tumorigenesis, neoangiogenesis and metastasis through different mechanisms [18–20,85]. For example, Ibrahim *et al.* [20] have shown that MDSCs play a critical role in colitis-associated colon tumorigenesis. High levels of MDSCs and IL-10 are detected in inflamed colon tissues, with the primary source of IL-10 being the MDSC population that had accumulated in these tissues. Of note, accumulation/homing of MDSCs to inflammation sites could be driven by C-X-C motif chemokine ligand 1/C-X-C motif chemokine receptor type 2 (CXCL1/CXCR2) [22,86]. In normal and cancerous colon tissues, IL-10 was shown to upregulate the expression of deoxyribonucleic acid (DNA) methyltransferase 1 (DNMT1) and DNMT 3 beta (DNMT3b) proteins in colon epithelial cells upon activation of STAT3, as the activated STAT3 binds to the promotor regions of the genes of these proteins and induce their expression. Subsequently, DNMT1 and DNMT3b proteins hyper-methylate the promotor region of the anti-tumour gene *irf8* resulting in interferon regulatory factor 8 (IRF8) protein downregulation. DNA methylation is an important mechanism that colorectal tumours harness to promote tumorigenesis. The latter study has provided a mechanistic pathway in which MDSCs directly take part in promoting tumorigenesis. Data from other studies have also provided evidence of other mechanisms by which MDSCs promote colorectal tumorigenesis. For example, increased C–C motif chemokine ligand 2 (CCL2) expression at the tumour site as a consequence of the accumulation of MDSCs in colorectal cancer tissues, at least in part [21], may lead to an elevation of type-2 immune response and thus, in theory, an elevation in the levels of IL-4 and IL-13 would be expected. Recently, IL-4 and IL-13 and their receptors (IL-4R and IL-13R) have been shown to be increased in colorectal cancer tissues, and this elevation was also shown to be associated with local metastasis [87]. Indeed, IL-4 and IL-13 are known to mediate the activation of STAT6 signalling pathway [88]. Jayakumar *et al.* [89] have reported that signalling through IL-4/IL-13-STAT6 pathway promotes intestinal tumorigenesis in Apc^Min/+^ mouse model, and provided evidence for a possible role of MDSCs. In their study, activation of STAT6 was essential for tumorigenesis and promoting the expansion and accumulation of MDSCs, and this could be considered a negative feedback loop. In another example, Yan *et al.* [23] showed that receptor-interacting protein kinase 3 (RIPK3) deficient MDSC that had accumulated in colorectal cancer tissues play a critical role in colorectal carcinogenesis. In brief, the reduction of RIPK3 expression in MDSCs resulted in increased cyclooxygenase-2 (COX-2)-transcription through the activation of nuclear factor kappa-light-chain-enhancer of activated B cells (NF-κB) pathway, with COX-2, in turn, catalysing the production of PGE2. Besides the ability to inhibit cytotoxic T cell responses, PGE2 suppressed RIPK3 expression in MDSC and colorectal cancer cells and induced NF-κB/COX-2 and expression of arginase 1 (ARG-1). Furthermore, PGE2 production at the tumour microenvironment could also increase the infiltration of MDSCs to the colorectal cancer tissues by increasing the secretion of CXCL1 which elicits its effects by signalling through its receptor CXCR2 resulting in the recruitment of MDSCs to the tumour microenvironment [22]. This signalling circuit is reported to accelerate colorectal cancer tumorigenesis, since targeting this circuit significantly attenuated colorectal tumour growth [23]. Still, other possible mechanisms have also been described in the literature. For example, upon recruitment to the tumour site MDSCs could also promote tumour growth and metastasis by secreting the inflammatory proteins S100 calcium-binding protein A8/A9 (S100A8/A9) which mediate the activation of mitogen-activated protein kinase (MAPK) and NF-κB signalling pathways in tumour cells [90–92]. Besides that, S100A8/A9 can enhance the immunosuppressive activity and regulate the accumulation of MDSCs at the site of inflammation [93], indicating that such proteins could play a major role in the pathogenesis of cancer, or at least certain cancer types. It is thus of central importance to realize that several molecular mechanisms could be involved in the tumorigenesis of a single cancer type, reflecting the complexity of the mechanistic pathways of tumorigenesis.

## 4. MDSC/IL-10, mechanisms of immune suppression in different pathological conditions

With respect to the molecular mechanisms of immune suppression by MDSCs, it is now well-established that there are different pathways by which these cells can mediate their immunosuppressive effects and more than one of these pathways (but not all) could be activated simultaneously to mediate their immunosuppressive effects [81]. It is thus rational to conclude that the mechanism(s) of immune suppression mediated by MDSCs is/are governed by different factors which include but are not limited to: (i) the type of MDSCs expanded, taking into consideration that M-MDSCs exert their immunosuppressive effects in a manner different from PMN-MDSCs; (ii) the pathological condition (i.e. cancer, infection, autoimmune diseases, allergic reactions, etc.); (iii) the stage of disease progression; as well as (iv) the inflammatory microenvironment and the anatomical site (e.g. peripheral blood, bone marrow, lymph nodes, spleen, etc.) where the immune suppression process is activated/triggered [81].

Secretion of immunosuppressive molecules is now considered to be a key mechanistic pathway to suppress various immunological responses. For example, MDSCs secrete copious amounts of immunosuppressive cytokines for example, IL-10 and TGF-β. It is essential to keep in mind that each of the latter cytokines could also express their immunosuppressive effects in different ways, according to the pathological condition and the inflammatory microenvironment. Since TGF-β is out of the scope of this work, the following discussion will be restricted to IL-10 only.

### 4.1. Cancer

It is clear that IL-10 is involved in the pathogenesis of different inflammatory pathological conditions, including several types of cancers. This does not mean that we should ignore the coexistence of other factors that could also be involved in the pathogenesis of inflammatory conditions including cancer. For example, a study on patients with anaplastic thyroid carcinoma has revealed that MDSC expansion is associated with disease progression and a positive association between the production of IL-10 and increased circulating MDSCs has also been reported, although the mechanism of action has remained undetermined [94]. Based on the fact that increased levels of IL-10 have been reported in many types of cancer, Hart *et al.* [28] conducted a study to determine the source of IL-10 elevation in tumour-bearing hosts in a murine model of ovarian cancer. In their study, MDSCs were shown to be the primary producers of IL-10 in the ascites of these mice [28]. This is consistent with the finding that depletion of MDSCs results in a remarkable reduction in IL-10 levels, and concomitantly, better outcomes leading to inhibition of tumour progression [95,96]. Hart and colleagues [28], showed that IL-10 dictates the immunosuppressive phenotype and function of MDSCs in the tumour microenvironment through interacting with IL-10-receptor (IL-10R) expressed on MDSCs, indicating that MDSC-derived IL-10 expresses autocrine effect on MDSCs themselves. Hart and colleagues have also pointed to the possibility that IL-10 could stimulate the development/emergence of T populations that express lymphocyte-activation gene 3 (LAG-3) molecules, given that the functional IFN-γ production is impeded in LAG-3^+^ T cells but not in LAG-3^−^ T cells. This indicates that IL-10 secretion by MDSCs results in the expression of paracrine effects on T cell function and phenotype.

In patients with ovarian cancer, a significant increase in MDSCs (particularly M-MDSCs) was also observed in different body compartments, including the ascites and peripheral blood, with a particular increase in the ascites, and the levels of M-MDSCs were shown to be directly correlated with poor prognosis [97]. The expansion of M-MDSCs in these patients was shown to be driven by IL-6 and IL-10 through their downstream STAT3 signalling pathway, as blocking either the IL-6 or IL-10 using neutralizing anti-IL-6 or IL-10 antibodies individually significantly reduced the expansion of ascitic fluid M-MDSCs [97]. Lamichhane *et al.* [98] have also shown that blocking IL-10 resulted in a remarkable decrease in infiltration of MDSCs to the ascites, indicating that there could be a positive feedback loop between MDSCs and IL-10. However, inhibition of STAT3 signal activation as well, significantly abrogated the expansion of ascitic fluid M-MDSCs to a level similar to that achieved by applying the two neutralizing anti-IL6/IL-10 antibodies in combination [97]. M-MDSCs from peripheral blood and ascitic fluid were able to restrict the proliferation of autologous CD4^+^ and CD8^+^ T cells and suppress the production of the effector cytokines IL-2 and interferon- *γ* (IFN-γ). This is of significance given that these adaptive immune cells and their secreted cytokines are essential for anti-tumour immunity. It is noteworthy that the suppressive effects of M-MDSCs on these adaptive immune cells were primarily mediated by ARG1 and inducible nitric oxide synthase (iNOS), both of which are expressed in response to STAT3 activation. Another suppressive mechanism through which IL-10 could take part in the pathogenesis of ovarian cancer, is through mediation of the expression of the immune check-point inhibitor ‘PD-1' in a STAT3-dependent manner [98]. Furthermore, high levels of IL-10 at the ascites of ovarian cancer patients was shown to increase the migration of tumour cells [99], thereby enhancing tumour progression.

To further support the importance of IL-10 in immunosuppression mediated by MDSCs, another study showed that MDSCs isolated from histone-deacetylase 11 (a negative regulator of IL-10 transcription in myeloid cells of both humans and mice) knock-out tumour-bearing mice express more immunosuppressive effects and were able to secrete greater amounts of IL-10 than that of the ‘wild type' tumour-bearing mice [100]. Parker and co-workers have shown that alarmin high mobility group box 1 (HMGB1) enhances immune suppression in tumour-bearing mice by facilitating the differentiation and suppressive activity of MDSCs [101]. In part, HMGB1 enhances the suppressive activity of MDSCs by enhancing their capacity to produce IL-10 which, in turn, suppresses anti-tumour immunity and polarizes the activation of pro-tumour immune phenotype.

In the serum of patients with B-cell non-Hodgkin lymphoma the levels of IL-10 are increased, and based on *in vitro* studies Xiu *et al.* [69] have shown that IL-10 is the underlying cause of M-MDSC expansion in these patients. Moreover, treatment of monocytes from B-cell non-Hodgkin lymphoma patients with IL-10 has been shown to decrease human leucocyte antigen–DR isotype (HLA-DR) levels and increase their suppressive capabilities [69]. Given that M-MDSC expansion and increased IL-10 production, in these patients, are directly associated with disease progression [102–106], this suggests a positive feedback loop between the expansion of MDSCs and IL-10 production.

MDSCs were also shown to play a central role in inhibition of tumour immunity in non-small cell lung cancer patients. This was, at least in part, through production of high levels of IL-10, at different anatomical sites including peripheral blood, lymph nodes and tumour tissues with a positive correlation between MDSC/IL-10 levels and disease progression [107]. The mechanism of T cell inhibition by MDSCs in non-small cell lung cancer patients was partially mediated by upregulating the expression of ARG1 through IL-10 production [43,107].

In gastric cancer, it has been also been observed that the expansion of MDSCs is associated with disease progression [108]. Importantly, Li *et al.* [108] have shown that vasoactive intestinal peptide (a novel cytokine) can drive CD14^+^ mononuclear cell reprogramming into M-MDSCs, possibly leading to MDSC expansion in gastric cancer patients. The immunosuppressive effect of these M-MDSCs on CD4^+^ T cells was found primarily to be due to the increased IL-10 production, which in turn, inhibited anti-tumour immune responses, through inhibiting the production of IFN-γ and IL-12, and therefore enhancing tumour growth. A similar scenario was also observed in prostate cancer in which IL-10 was responsible for the immune suppressive effects on T cells mediated by M-MDSCs [109].

From another point of view, PD-L1 overexpression on MDSCs under hypoxic conditions is observed, and its expression at both the mRNA and protein levels was shown to be regulated by hypoxia-inducible factor 1-alpha (HIF-1*α*), through a direct binding to the hypoxia-response element (HRE) in the PD-L1 proximal promoter [110]. Furthermore, hypoxia enhanced the immunosuppressive activity of MDSCs. Although the major goal of the study of Noman and his colleagues [110] was to determine the impact of hypoxic environment on the expression of immune checkpoint receptors on MDSCs, some important observations regarding IL-10 were reported [110]. For instance, MDSCs cultured under hypoxic conditions were shown to produce significantly higher levels of IL-6, IL-10, and TGF-β, but not IL-12, when compared to the normoxia condition. Interestingly, blockade of PD-L1 on MDSCs under hypoxia, but not normoxia, resulted in a significant reduction in IL-6 and IL-10 secretion, but not TGF-β, and this event was accompanied by a significant attenuation of the suppressive activity of MDSCs, suggesting a possible role of these cytokines in this process. Noman *et al*., [110] found that blocking of IL-10 but not IL-6, by neutralizing antibodies resulted in the attenuation of the suppressive immune responses of MDSCs exerted on specific and non-specific cytotoxic T cells under hypoxic condition. These findings further support the importance of IL-10 in the immunosuppressive activity of MDSCs. Under certain circumstances, it has been observed that interaction of MDSCs with T cells could enhance the production of IL-10 by T cells [111]. IL-10, in turn, promotes STAT3 activation in MDSCs, which results in upregulation of the expression of PD-1 on MDSCs, and PD-1^+^ MDSCs can suppress immune responses by a mechanism involving the expression of ARG-1 and indoleamine 2,3-dioxygenase (IDO) [111]. Taken together, these data show the complexity of the interconnections between IL-10 and different mechanisms of immunosuppression by MDSCs.

### 4.2. Sepsis and infection

During sepsis in mice, MDSCs accumulate in the late or chronic phase and both the pro-inflammatory cytokine IL-6 and the anti-inflammatory cytokine IL-10 play a critical role in the mediation of MDSC expansion. The activation of STAT3/S100A9 pathway, is thought to be one mechanism that allows MDSC expansion. Indeed, both IL-6 and IL-10 can activate naive immature myeloid cells to become MDSCs through inducing the expression of S100A9 upon STAT3 phosphorylation. Of note, phosphorylation of STAT3 is known to induce the expression of S100A9 in immature murine myelocytes [61]. Interestingly, Bah *et al.* [112] have concluded that IL-10, but not IL-6, is responsible for MDSC expansion during late sepsis in mice. This is because a swift increase in the level of IL-6 was observed during the early phase (1–5 days) of sepsis in both the wild-type and S100A9 knock-out mice, and treatment with anti-IL-6 neutralizing antibodies was very efficient at decreasing the expansion of MDSCs in the early septic phase only. By contrast, a continuous and gradual increase in the levels of IL-10 was observed in the wild-type mice especially after 6 days of the onset of sepsis, while a slight elevation in IL-10 was observed in S100A9 knock-out mice during the early septic phase only. This may explain why blocking IL10 was so efficient in reducing the accumulation of MDSCs only in late sepsis. One important finding in this study is that, of the two cytokines only the IL-10 was able to promote re-localization of cytosolic S100A9 of naive MDSCs to the nucleus and activate immature myeloid cells to the immunosuppressive phenotype. Furthermore, silencing the S100A9 in MDSCs during late sepsis abolished their immunosuppressive capability, indicating that the intracellular but not the extracellular S100A9 was responsible for activating immunosuppressive MDSCs.

Alkhateeb *et al*. [113] have also confirmed the results of Bah *et al*. [112] and highlighted the importance of IL-10 in driving MDSC expansion and activation of the immunosuppressive phenotype in late/chronic sepsis. They report that IL-10 works by activating STAT3 and mediating the translocation of S100A9 from the cytosol to the nucleus where it triggers the expression of certain microRNAs (namely miRNA-21 and miRNA-181b), possibly by stabilizing the phosphorylated-STAT3-C/EBP*β* complex and facilitating the binding of this complex to the miRNA promoter region [113]. The increased expression of these microRNAs in MDSCs results in increased expression of NFI-A (a myeloid differentiation-related transcription factor) that lead to the expansion of immunosuppressive MDSCs during late sepsis [113].

Infection with *Staphylococcus aureus* (*S. aureus*) is known to promote the expansion of MDSCs also [114]. Such expansion was shown to be associated with the persistence of *S. aureus* as a consequence of inhibition of specific T cell responses against this pathogen. In mice infected with *S. aureus*, MDSCs inhibited monocyte/macrophage-mediated anti-bacterial immunity by producing high levels of IL-10, thus leading to infection persistence [115]. In patients with chronic hepatitis B virus (HBV) infection, MDSC expansion is reported to be directly associated with the viral load in plasma of HBV infected patients. MDSCs were able to suppress adaptive immune responses, in particular ‘specific T cell responses', against HBV via PD-1/IL-10 [42]. Indeed, MDSCs from these patients produce significantly greater amounts of IL-10 than that of healthy subjects (i.e. *ex vivo* studies indicated that MDSCs from HBV patients could produce IL-10 in a spontaneous manner, while in contrast, healthy MDSCs cannot do so). Notably, the suppressive immune responses on T cells in the case of HBV infection was exclusive to IL-10 secretion, and this event was dependent on PD-1 stimulation on MDSCs, but not PD-LI, indicating that an interrelation between PD-1 and IL-10 exists [42].

### 4.3. Autoimmunity

Indeed, recent investigations have shown that MDSCs could play a critical role in autoimmune disorders (for example, rheumatoid arthritis (RA), inflammatory bowel disease, type 1 diabetes, multiple sclerosis, autoimmune hepatitis, alopecia areata, eczema and systemic lupus erythematosus) [50,51,116–119]. However, there is an inconsistency in results regarding their role in such pathological conditions. Some investigators have shown that MDSCs play a good (yang) role in controlling the disease progression [9,120–123], while others have contradicted this view, i.e. MDSC play a bad (yin) role in disease progression [73,74,124]. To solve such perplexing results, we have recently established the yin–yang law of MDSC elsewhere [15]. In brief, we have stated that if MDSCs are naturally expanded in a pathological condition without any external intervention (adoptive cell transfer or mediating the expansion of MDSCs by treatments) and such expansion was accompanied by any improvement in the clinical status of that pathological condition, then such expansion should be considered as a yang face. Otherwise, if the natural expansion of MDSCs was not accompanied by any improvement in the clinical status of or it was associated with any negative impact on a given pathological condition, then such expansion should be considered as an unwanted (yin) consequence. On the other hand, this does not mean to exclude the possibility that MDSCs could be harnessed to control inflammation under certain circumstances, where such cells are not expanded or the level of expansion does not reach the level that allow them to control inflammation. To prove such a claim, in a brief manner, we sought to address the role of MDSCs in RA, an example of autoimmune disorders. Although several studies have indicated that MDSCs are effective in controlling the disease progression in a collagen-induced arthritis (CIA) mouse model [9,120–123], these studies did not investigate the impact of natural expansion of MDSCs on disease progression in an RA animal model; rather, they investigated the impact of adoptively transferred MDSCs in controlling RA in CIA mouse model. We believe that such experimental condition cannot be used to determine the impact of MDSCs on disease progression. This is true for two reasons: first, the differences in RA disease progression scenario between human and CIA mouse model; second, the absence of data addressing the role of MDSCs in RA in humans. It is worthy to note that the preclinical studies on animal models do not necessarily represent exactly the same clinical status in humans. Therefore, investigating the role of natural expansion of MDSCs in autoimmune disorders (such as RA) in animal models and humans is needed, so that a balanced comparison can be made.

Therefore, at this time point, a definitive conclusion regarding the role of MDSCs/IL-10 in autoimmune disorders cannot be made. Hence, additional investigations on animal models as well as humans are required to determine whether MDSCs/IL-10 are good or not.

### 4.4. Allergy

In asthma (a chronic inflammation of airways), an imbalance between CD4^+^ T helper cell subsets, namely Th1 (‘type 1 immunity') and Th2 cells (‘type 2 immunity’) with a shift towards Th2 cells is induced. Cytokines play an indispensable role in this shift during asthma in both humans as well as animal models. IL-12 is critical for the function and guiding of the differentiation of Th1 cells. By contrast, IL-10 is known to participate in guiding the differentiation of Th2. Therefore, the two cytokines are involved in the pathogenesis of asthma. Neutralization of IL-10 has been shown to reverse this shift. MDSCs are a primary source of IL-10, therefore studying the association between MDSC/IL-10 levels in asthma is important. In this regard, Zhang *et al.* [125] have shown that the levels of IL-10 and MDSC dramatically increase during asthmatic children and mice. This may indicate that MDSCs and IL-10 could play a role in the pathogenesis of asthma, suggesting additional investigations.

### 4.5. Ageing

Accumulation of MDSCs over time in aged individuals has been shown to increase the incidence of tumour development [126]. For instance, Chen and others have reported that MDSCs promote age-related increase of lung cancer growth via inhibiting anti-tumour T cell responses through upregulating the expression of immune checkpoint protein, namely PD-1 (the so-called B7-H1). Different cytokines could play a role in upregulating the expression of PD-1 including IL-10, granulocyte-macrophage colony-stimulating factor (GM-CSF) and IFN-γ [127,128]. Interestingly, IL-10 was responsible for the stimulation of PD-1 expression in MDSCs as the inhibition of IL-10 by neutralizing anti-IL-10 antibody was associated with a significant reduction in PD-1 expression in MDSCs [126], supporting the interrelation between PD-1 and IL-10.

Taken together, these studies clearly emphasize that MDSCs/IL-10 play a direct indispensable role in the pathogenesis of various pathological conditions. However, it is important to remember that additional investigations are required to address their role in other pathological conditions such as autoimmune disorders and other inflammatory conditions.

## 5. MDSCs/IL-10 and cross-talking with other immune cells

**Figure 1.**
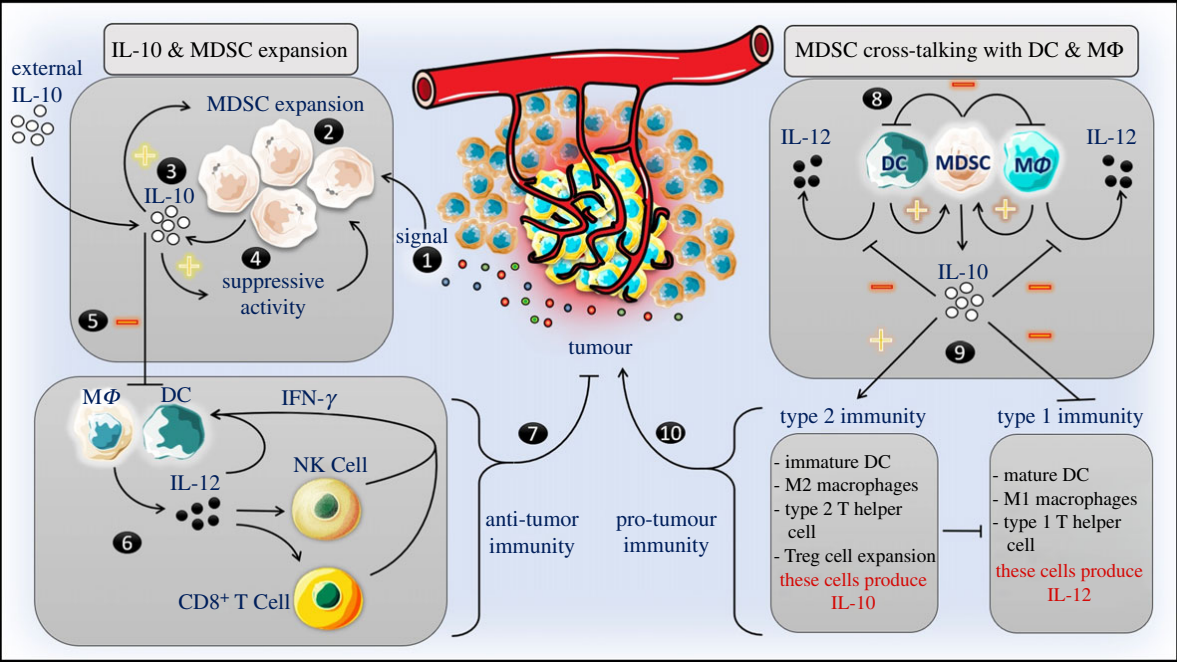
Mechanisms of anti-tumour immune response subversion by MDSC/IL-10. In fact, inflammation provides a signal that promotes MDSC expansion. MDSCs are primary producers of IL10 a potent antiinflammatory cytokine. In turn, MDSC-derived IL-10 and IL-10 produced by other cells could also promote the expansion of MDSC and enhance the immunosuppressive activity. IL-10 can also inhibit the production of IL-12 by dendritic cells (DCs) and macrophages (M*Φ*). IL-12 is essential for initiation of anti-tumour immunity by activating the maturation of natural killer (NK) cells that, in turn, produce inflammatory cytokines such as IFN-γ that further activate the maturation of professional APCs such as DCs and M*Φ* triggering a positive feedback loop. In addition, IL-12 can activate cytotoxic T lymphocytes (CD8^+^ T cells). NK cells and CD8^+^ T cells are essential to fight tumor cells. As such, MDSCs can inhibit antitumor immunity at least in part through production of copious amounts of IL-10, i.e. without a direct cell-to-cell contact. On the other hand, direct cross-talking between MDSCs and DCs and/or M*Φ* can also inhibit the maturation of DCs and blunt the production of IL-12. Rather, this direct cell contact can potentiate the production of IL-10 by MDSCs, which in turn further inhibits the production of IL-12, an essential driver of type 1 immunity ‘anti-tumour immunity’. MDSCs/IL-10 can inhibit type 1 immunity while promote the type 2 immunity that is known to promote tumour progression.

From another point of view, MDSCs can also inhibit the anti-tumour immunity by cross-talking with other immune cells (figure 1). For instance, the Rosenberg group [16] showed that MDSC subvert innate and adaptive anti-tumour immunity by synergizing the production of IL-10 in tumour-bearing mice and cell cultures, after skewing the phenotype and the function of M*Φ* to the alternatively activated M*Φ* (M2) rather than the classically activated M*Φ* (M1). For this event to be accomplished, they have shown that a direct cell-to-cell (MDSCs–M*Φ*) contact is required. Unlike the anti-tumour M1, M2 express high levels of IL-10 and low levels of IL-12, and are known to enhance tumour progression [16,129,130]. The increased IL-10 production was exclusive to MDSC upon cross-talking with M*Φ* but not as a result of M1 to M2 shift, because this occurred when MDSCs cross-talked with IL-10^−/−^ M*Φ*, while the observed IL-12 reduction was due to the shift of M1 to M2 [16]. These results were also further confirmed by the Rosenberg group later [17], and they also reported that MDSC IL-10 downregulates the production of IL-6 while increasing the production of NO by M*Φ* [17]. Indeed, MDSCs cross-talking with M*Φ* result in activation of an intermediate M*Φ* state that share some properties of both M1 and M2, i.e. these M*Φ* produce low levels of IL-12 and IL-6 ‘like M2', while expressing high levels of NO ‘like M1'. Furthermore, Zhou *et al.* [131] reported that MDSC can suppress anti-lung-tumour immune responses by shifting the inflammatory (type 1 immunity) towards the anti-inflammatory immune responses (type 2 immunity). MDSC suppression of anti-lung-tumour immune responses occurred through inhibiting the maturation of DCs, directing the polarization of M*Φ* to the M2 phenotype, and mediating the expansion of Treg cells. Similarly, other investigators have shown that increased regenerating islet-derived protein 3 gamma (Reg3 g) expression in pancreatic ductal adenocarcinoma is associated with tumorigenesis of this cancer by promoting the recruitment of immune regulatory cells, including MDSCs, to the tumour microenvironment. Reg3 g-recruited MDSCs can inhibit the maturation of DCs, inhibit the effector function of cytotoxic T cells, and shift the type 1 immunity towards the type-2 immunity [132]. In liver cancer, MDSC expansion was shown to inhibit the function of T cells and lead to tumour progression by inhibiting the activity of different innate immune cells, including NK cells and DC [133–135]. Hu and colleagues [133] have demonstrated that MDSC expansion can lead to hepatocellular carcinoma development in mice through inhibition of the T-cell stimulatory activity of DC and inhibition of IL-12 production by the IL-10 that was produced by MDSCs [133]. Indeed, elevation of IL-10 secretion by MDSC or M2 at the expense of reduction of IL-12 secretion by M2 and/or inhibition of IL-12 producing cells, namely DCs, can affect the immune responses mediated by different immune cells (including NK cells and DC, as well as T cells) and shifts the inflammatory responses to the anti-inflammatory response. Of note, IL-12 is essential for NK cell activation and production of tumour necrosis factor-alpha (TNF-α) and IFN-γ [2], as well as for the subsequent activation of anti-tumour immune responses, given that NK cells are the principal innate effector cells that express potent anti-tumour immunity. On the other hand, high levels of IL-10 can inhibit the maturation of DC which are the most professional antigen-presenting cell (APC) and a major IL-12 producing cell among the entire immune cells. This, in turn, can inhibit the activation of specific anti-tumour/anti-pathogen T cells responses and hamper the activation of NK cells, respectively. Inhibition of NK cells can also affect the maturation of DC, since it is known that NK cells cross-talk with DCs, either directly via cell-to-cell contact and/or indirectly via cytokines network, and this cross-talking has mutual reciprocal effects [2]. In line with these data, Choi *et al.* [136] have shown that treatment of tumour-bearing mice with IL-12 can improve the anti-tumour immune responses by downregulating the suppressive function of MDSCs, decreasing M2 numbers, and increasing the proportion of APCs (including mature DC) by guiding the differentiation of M-MDSCs to APCs. It has also been shown that MDSCs can inhibit anti-tumour immune responses by driving the expansion of Treg cells in a cell-to-cell contact independent manner, at least in part, by inducing the secretion of IL-10 upon activation of STAT3 pathway [131]. These Treg cells can inhibit cytotoxic T cells via immune check-point inhibitors, such as PD-1 (also known as CD274 or B7-H1) and CTLA-4.

On the other hand, once activated, MDSC could also participate in reprogramming of mature monocytes to become M-MDSCs or MDSC-like cells, at least in part, through IL-10 [70,130], initiating a negative feedback loop and thus entering a vicious circle. Interestingly, co-incubation of monocyte-derived DCs (moDCs) with IL-10-treated APC (MDSC-like cells; which are generated from human monocytes under the differentiation condition of moDC and treated with IL-10 in the presence of IL-4 and GM-CSF) results in a reduced capacity to stimulate the activation of allogenic T cells [130]. Perhaps this is because these cells express different classes of immune suppression molecules such as PD-1, glucocorticoid-induced tumour-necrosis-factor-receptor-related-protein (GITR) and its ligand GITRL, as well as syndecan-4, indicating that these cells themselves are immunosuppressive. Further, co-incubation of MDSC-like cells with mature and immature moDC resulted in a substantial increase in the expression of the inhibitory molecules GITR, PD-1, PD-L1 and osteoactivin on mature moDC, as well as GITR and PD-1 on immature moDC, indicating that MDSC-like cells can affect the surrounding cells. Moreover, treatment of healthy MDSCs with IL-10 significantly increased their suppressive capacity, in part through inducing the expression of GITR, GITRL, osteoactivin, syndecan-4 and PD-L1 [130], supporting the notion that healthy MDSCs are much less suppressive than pathologically activated MDSCs.

Lamichhane *et al.* [98] have also reported that IL-10 in the ascites of ovarian cancer-bearing mice can increase the expression of PD-1 and PD-L1 on tumour infiltrated DCs as well as the bone marrow-derived DCs. Expression of PD-1 on DCs, in turn, impairs their function; since it has been shown that elevation of PD-1 expression on DCs maintains them in a suppressive phenotype [137,138]. IL-10-treated DCs can then suppress anti-tumour adaptive immunity by inhibiting T cell responses upon ligation of PD-1 on T cells with its ligand ‘PD-L1' on DCs [98]. Although these investigators did not explore the mechanism by which IL-10 is increased in the ascites, one possible mechanism could be due to the increased infiltration of MDSCs, which are considered to be among the major IL-10-producing cells. This could be a plausible mechanism since the blocking of IL-10, but not PD-1, decreased the infiltration of MDSCs to the ascites. Interestingly, this study has shown that blocking the PD-1 by neutralizing antibodies was associated with increased IL-10 production by DCs and this did not result in a change in the immunosuppressive microenvironment of the tumour, indicating that IL-10 compensates for the PD-1 blockade. Also, this explains why some cancer types do not benefit from treatments that target PD-1/PD-L1-axis [98,139]. Targeting both IL-10 and PD-1 by neutralizing antibodies, but not PD-1 alone, resulted in reduced tumour burden, augmented adaptive anti-tumour immunity and thus enhancement in survival of tumour-bearing mice. Taken together, these data underscore the importance of IL-10 in immune suppression by MDSCs.

## 6. MDSC/IL-10 as a therapeutic target

As mentioned above, it is now clearly evident that MDSCs/IL-10 could be targeted to control different inflammatory conditions. In the context of cancer, MDSCs and IL-10 can inhibit anti-tumour immune responses, thereby leading to tumour progression. MDSCs are the primary producers of IL10 which, in turn, can limit the activity of helper T cells and cytotoxic T lymphocyte (CTL) against tumour cells by different mechanisms including decreasing the expression of major histocompatibility complex (MHC) class I on tumour cells [140]. Additionally, IL-10 can inhibit anti-tumour activity of NK cells, for example, by increasing the expression of non-classical MHC class I on tumour cells, given that non-classical MHC class I can inhibit NK cell activity [141]. On the other hand, IL-10 can limit the immune responses mediated by DCs (inhibit antigen presentation) via decreasing the expression of MHC class II, intracellular adhesion molecules (e.g. ICAM-1), and costimulatory CD80 and CD86 molecules via inhibiting production of IL-12 [142,143]. IL-10 can also trigger the generation and activation of Treg cells [144], all of which can limit anti-tumour immune responses, leading to cancer disease progression. It is thus not surprising to know that targeting IL-10 for inhibition in such conditions has been suggested as a therapeutic approach to enhance anti-tumour immune responses. For this reason, Rossowska *et al*. [145] reported that temporary elimination of IL-10 represents an effective way to decrease the immune suppression mediated by MDSCs and considered a useful approach to enhance the efficacy of immunotherapy for cancer. Reversing the immunosuppression of the tumour microenvironment has been suggested as a therapeutic approach to combat tumour growth. As such, Liu *et al*. [146] have reported that endostatin can achieve such goals in lung cancer by limiting the recruitment of MDSCs and decreasing the production of anti-inflammatory cytokines, including IL-10 among others. Consistent with this view, Tan *et al.* [147] have shown that depletion of MDSCs, particularly IL-10-producing PMN-MDSCs, which can inhibit DCs that activate anti-tumour cytotoxic T cell responses, can enhance the therapeutic efficacy of modified vaccinia Tiantan strain by inducing anti-tumour cytotoxic T cell responses in mesothelioma. Treatment with zoledronic acid impaired the accumulation of MDSCs to the tumour site resulting in delayed tumour growth rate, and increased recruitment of T cells to the tumour and prolonged the overall survival [148]. Indeed, this was associated with a reduction in IL-10 production and a shift towards type 1 response with increased IFN-γ production [148]. Similarly, Sinha and co-workers [149] have shown that treatment of tumour-bearing mice with WithaferinA (the most abundant constituent of the plant *Withania somnifera*) can reduce the accumulation of MDSCs at the tumour site. Additionally, they have reported that treatment of MDSCs with Withaferin A can minimize their capacity to produce IL-10 in a dose-dependent manner. Furthermore, they have shown that Withaferin A can prevent the increased production of IL-10 by MDSCs upon cross-talking with M*Φ*, therefore inhibiting pro-tumour immune responses, namely type 2 immune responses [149]. Likewise, targeting MDSCs/IL-10 directly or indirectly in other pathological conditions such as sepsis/infection [112,113], certain autoimmune disorders, and certain allergic disorders such as asthma [125] could also provide a promising approach to control such pathological conditions. However, since the role of MDSCs is less established in non-cancer pathological conditions, additional investigations are required.

MDSCs/IL-10 could also be indirectly inhibited by targeting regulators and transcription factors (such as STAT3) involved in MDSC expansion and IL-10 production. Activation of the STAT3 transcription factor signalling pathway by IL-10 is well documented in several pathological conditions. Furthermore, STAT3 activation is not only important for MDSC generation and accumulation, it is also important for their immunosuppressive function, since it regulates the expression of different molecules involved in immune suppression such as IDO-1 and ARG-1, as well as IL-10 and vascular endothelial growth factor (VEGF) [150–152]. This suggests that STAT3 could be considered as a potential therapeutic target [153–156]. However, to delineate the importance of IL-10 in the immunosuppressive effect mediated by MDSCs through STAT3, Hellsten and colleagues [157] have demonstrated that inhibition of STAT3 using galiellalactone prevented the generation of MDSC-like cells from treated prostate cancer *ex vivo*. They also observed a significant reduction in immunosuppressive activity of MDSCs, possibly as a result of a significant reduction in IL-10 secretion by MDSCs. Similarly, targeting STAT3 in other pathological conditions such as sepsis could also provide a therapeutic avenue to control the expansion of MDSCs and regulate the production of IL-10, and thus, enhance immune responses.

Targeting ST100A8/A9 pathways in certain pathological conditions, wherever these pathways are involved in MDSC expansion and IL-10 production as aforementioned, is also another strategy to target MDSCs/IL-10. Still, targeting HMGB1 or histone-deacetylase 11 by specific inhibitors could also provide an avenue to inhibit MDSC expansion and limit IL-10 production and thus enhance anti-tumour/infection immune responses.

## 7. Conclusion

It has become clearly evident that MDSCs are an important immunoregulator cell population of the immune system which seems to be activated mainly in the setting of pathological conditions. Furthermore, the information available about their role under normal conditions is inadequate. Together, this explains why MDSCs are often described as pathologically activated cells. Although the role of MDSCs relatively is well studied in oncological studies, their pathological role in other pathological conditions is still in its infancy, explaining why investigators have emphasized on results obtained from cancer studies. This necessitate additional investigations on other chronic inflammatory conditions as well as infectious diseases.

With respect to their immunosuppressive function, MDSCs express their activities through different mechanisms. Secretion of copious amounts of immunoregulatory cytokines such as IL-10 and TGF-β is considered to be a major mechanism by which MDSC can downregulate immune responses. The purpose of this work was to address the mechanisms of immunosuppression mediated by MDSCs/IL-10 and their role in the pathogenesis of different inflammatory conditions according to the available data in the literature. Indeed, IL-10 can mediate and regulate immune responses at the cellular and molecular levels. Several studies have shown that there is a positive feedback loop between production of IL-10 and the expansion of MDSCs. In other words, MDSCs are among the primary producers of IL-10 which, in turn, positively mediate the expansion and recruitment of MDSCs to the inflammatory site. Intriguingly, regulation of MDSC expansion by IL-10 and the production of this anti-inflammatory cytokine are governed by different mechanistic pathways, explaining why there are several targets that could be used to control the expansion of MDSCs and the production of IL-10.

It is essential to realize that IL-10 can directly inhibit immune responses by upregulating the expression of cell membrane suppressor molecules such as PD-1 and PD-L1 on MDSCs. This, in turn, can suppress immune response activation upon direct cross interaction with adjacent immune cells that express corresponding ligands at the inflammatory site. On the other hand, IL-10 can suppress immune responses indirectly by shifting inflammatory to anti-inflammatory immunity by inhibiting the maturation and activation of innate immune cells such as DC, M*Φ* and NK cells, while triggering the expansion of Treg cells, both of which can lead to inhibition of specific immune responses against abnormal (tumour and/or infected) cells, resulting in disease persistence.

## Data accessibility

This article has no additional data.
